# Loss of *Stat3* in Osterix^+^ cells impairs dental hard tissues development

**DOI:** 10.1186/s13578-023-01027-1

**Published:** 2023-04-23

**Authors:** Laiting Chan, Jiarui Lu, Xin Feng, Lichieh Lin, Yichen Yao, Xiaolei Zhang

**Affiliations:** 1 Guangdong Provincial Key Laboratory of Stomatology, Guanghua School of Stomatology, Hospital of Stomatology, Sun Yat-sen University, Guangzhou, Guangdong China; 2 Department of Stomatology, The Eighth Affiliated Hospital, Sun Yat-sen University, 3025 Shennan Middle Road, Shenzhen, 518033 Guangdong China

**Keywords:** Stat3, Dental mesenchymal cells, Dental hard tissues, HERS, Conditional knockout mice

## Abstract

**Background:**

Mutations in the signal transducers and activators of transcription 3 (*STAT3*) gene result in hyper-IgE syndrome(HIES), a rare immunodeficiency that causes abnormalities in immune system, bones and teeth. However, the role of Stat3 in development of dental hard tissues was yet to investigate.

**Methods:**

In this study, a transgenic mouse of conditional knockout of *Stat3* in dental mesenchymal cells (*Osx*-*Cre*; *Stat3*^*fl/fl*^, *Stat3* CKO) was made. The differences of postnatal tooth development between control and *Stat3* CKO mice were compared by histology, µCT and scanning electron microscopy.

**Result:**

Compared with the control, *Stat3* CKO mice were presented with remarkable abnormal tooth phenotypes characterized by short root and thin dentin in molars and incisors. The enamel defects were also found on mandibular incisors. showed that Ki67-positive cells significantly decreased in dental mesenchymal of *Stat3* CKO mice. In addition, β-catenin signaling was reduced in Hertwig's epithelial root sheath (HERS) and odontoblasts of *Stat3* CKO mice.

**Conclusions:**

Our results suggested that Stat3 played an important role in dental hard tissues development, and Stat3 may regulate dentin and tooth root development through the β-catenin signaling pathway.

## Background

Teeth are mineralized organs composed of three unique hard tissues, enamel, dentin, and cementum, and supported by the surrounding alveolar bone. Reciprocal interactions between epithelial and mesenchymal play a fundamental role in the development of dentin and enamel [1, 2]. During the bell stage of tooth development, the inner enamel epithelium signals to dental mesenchymal cells and induces odontoblast differentiation. After the odontoblasts have been induced to differentiate, they signal back to the epithelium and induce their differentiation into ameloblasts [3]. Teeth and bone tissues have similar physiological and biochemical properties. In the case of skeletal metabolic disturbances, tooth mineralization is susceptible to similar developmental defects as bone [4]. For example, vitamin D-deficient rickets can cause defects in both skeletal and dental mineralization [5].

Signal transducers and activators of transcription 3 (Stat3) is a member of the Stat family. It is widely expressed in a variety of cells, mediating cell proliferation, differentiation and apoptosis, and is involved in physiological processes such as self-renewal of embryonic stem cells [6]. Stat3 in skeleton is important for the maintenance of intraosseous homeostasis and bone differentiation [7–9]. Mutations in *STAT3* result in hyper-IgE syndrome (HIES), a rare primary immunodeficiency [10]. In addition to severe immune deficiencies, HIES patients exhibit skeletal and dental abnormalities, including osteoporosis, scoliosis, and recurrent pathological fractures [11], as well as delayed tooth eruption, multiple caries, and oral mucosal disease [12]. Our previous studies reported that loss of *Stat3* in mesenchyme exhibited skeletal malformations and mineralization abnormalities in mice [13, 14]. As some studies reported, Stat3 is expressed in dental epithelial cells and odontoblasts during tooth development [15, 16]. Bin Zhang *et al*. reported that loss of *Stat3* in epithelial cells resulted in delayed enamel mineralization in mouse incisors [16]. These studies suggested that Stat3 is important for the development of bone and teeth. The effect of Stat3 in the development of dental hard tissues deserved an interesting research question to explore.

In our current study, we established a mouse model in which Stat3 was conditional knockout (CKO) in dental mesenchyme using an Osterix (Osx/ Sp7) promoter driven by Cre recombinase. The dental hard tissues of *Stat3* CKO mice were compared with their Cre-negative littermate controls to investigate the role of Stat3 signaling in postnatal dental hard tissue development. The hypothesis of this study is that loss of Stat3 could lead to defects in tooth hard tissue formation.

## Materials and methods

### Animals

*Osx*-*Cre* and *Stat3*^*fl/fl*^ mice were purchased from Jackson Laboratory (*Osx*-GFP-*Cre*, stock no. 006361 [17] and *Stat3*^*fl/fl*^, stock no. 016923 [18]. In order to generate *Stat3* conditional knockout mice, firstly *Osx*-*Cre* mice were crossed with *Stat3*^*fl/fl*^ mice to obtain the offspring of *Osx*-*Cre*; *Stat3*^*fl/*+^ mice. Secondly, the *Osx*-*Cre*; *Stat3*^*fl/*+^ mice were mated with *Stat3*^*fl/fl*^ mice to have *Osx*-*Cre*; *Stat3*^*fl/fl*^ (hereinafter referred to as *Stat3* CKO) mice. *Stat3* CKO served as the experimental group and *Osx-Cre*^−^ littermates were controls. Mouse tail genotyping was performed by polymerase chain reaction (PCR). The animal experiments were reviewed and approved by the Animal Ethics and Welfare Committee of Sun Yat-Sen University (approval No. SYSU-IACUC-2021-000101).

### Histology and immunohistofluorescence

The mandibles of mice were dissected and fixed in 4% paraformaldehyde (PFA, ServiceBio, Wuhan, China) overnight. After the samples were decalcified by 0.5M EDTA (pH 8.0) solution and dehydrated by gradient sucrose, they were embedded into OCT and frozen sections were made with a thickness of 10 µm. Slides were stained with hematoxylin and eosin (H&E).

For immunohistofluorescence, sections were infiltrated with PBT (1 × PBS + 0.1% Tween 20) and blocked in 10% goat serum. Immunofluorescence staining was performed using standard methods. The information of primary and secondary antibodies were listed in Table 1. The images were captured by an inversion fuorescence microscope (Zeiss, Oberkochen, Germany).

**Table 1:** Antibodies for immunofluorescence

Antibody raised against	Dilution	Source (cat. No)
Primary antibody		
COL1A1 rabbit pAb	1:400	Abclonal (A1352)
KI67 rabbit mAb	1:400	CST (9129)
OCN rabbit mAb	1:200	Affinity (DF12303)
DMP-1 rabbit mAb	1:100	Affinity (DF8825)
β-catenin rabbit mAb	1:100	CST (8480)
Secondary antibody		
Anti-rabbit Alexafluor-594	1:400	Invitrogen
Anti-rabbit Alexafluor-488	1:400	Invitrogen

### µCT analysis

The mandibles of 3-week-old (3WK) and 8-week-old (8WK) female mice were dissected and stored in ethanol subject to µCT scanning (μCT50; Scanco Medical Ag, Bassersdorf, Switzerland), and subsequently reconstructed and analyzed with Materialise Mimics Innovation Suite Medical 21.0 software (Materialise, Leuven, Belgium).

### Scanning electron microscope (SEM) and energy dispersive spectrometer (EDS)

The mandibles of 8-week-old mice were fixed in 4% paraformaldehyde overnight, and then dehydrated and dried in gradient ethanol. After spray gold treatment, the samples were analyzed using scanning electron microscopy (ZEISS Sigma 300; Carl Zeiss AG, Jena, Germany) and the energy dispersive spectrometer (Bruker Quantax Xflash SDD 6).

### Statistical analysis

The quantitative data were presented as mean ± standard deviation (SD) of at least three independent samples. Student's t-tests were used to compare the difference between *Stat3* CKO and the control groups. The GraphPad Prism 8.0 software was applied for statistical analysis. The significance level was set at *P* < 0.05. Asterisks denotes the difference with statistical significance (***P < 0.001, ** *P* < 0.01, *P < 0.05).

## Results

### Cre-GFP expression in teeth

**Fig. 1:**
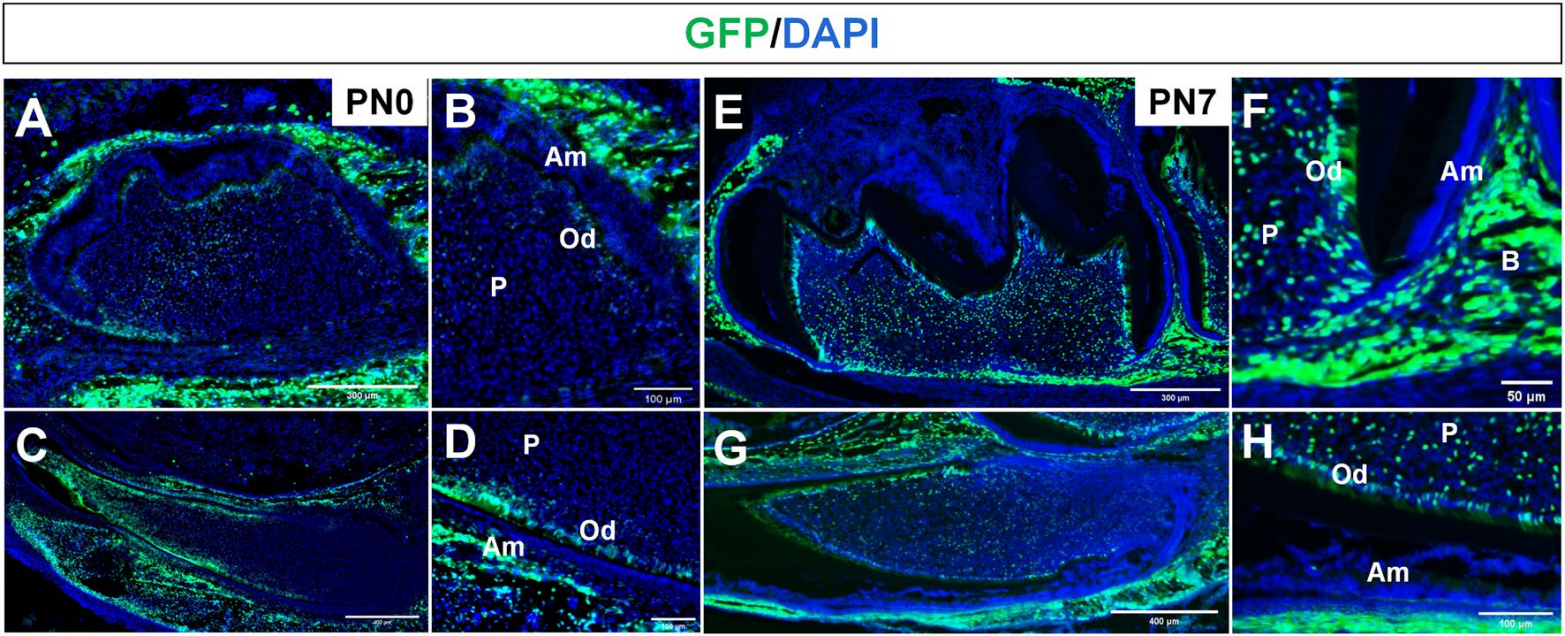
Cre-GFP expression in teeth.** A**–**D** Cre-GFP was detected as green fluorescence in alveolar bone and local odontoblasts in PN0 *Stat3* CKO mice. **E**–**H** At PN7, Cre-GFP was expressed in dental mesenchyme including odontoblasts and dental pulp. Cre was also seen in the alveolar bone. GFP signal was not detected in cells of epithelial origin including ameloblasts. *Am* ameloblasts, *Od* odontoblast, *P* pulp, *B* alveolar bone

The *Osx*-*Cre* mice expressed GFP-Cre fusion protein under the control of the Osx promoter [19, 20]. In this system, Osx-expressing cells exhibit green fluorescence. The Cre-GFP expressions in mandibular first molars and incisors were assessed. At postnatal day 0 (PN0) of *Stat3* CKO mice, Cre-GFP was expressed as green fluorescence in the alveolar bone at molars, incisors and local odontoblasts (Fig. 1A–D). At postnatal day 7 (PN7), Cre-GFP was expressed in a great majority of dental mesenchyme cells including odontoblasts and dental pulp (Fig. 1E–H). Cre was also seen in the alveolar bone, while dental epithelial cells such as ameloblasts and the Hertwig's epithelial root sheath (HRES) were not labelled. The results indicated that *Osx*-*Cre *was specific in dental mesenchymal.

### Abnormal enamel in the incisor of* Stat3* CKO mice

**Fig. 2:**
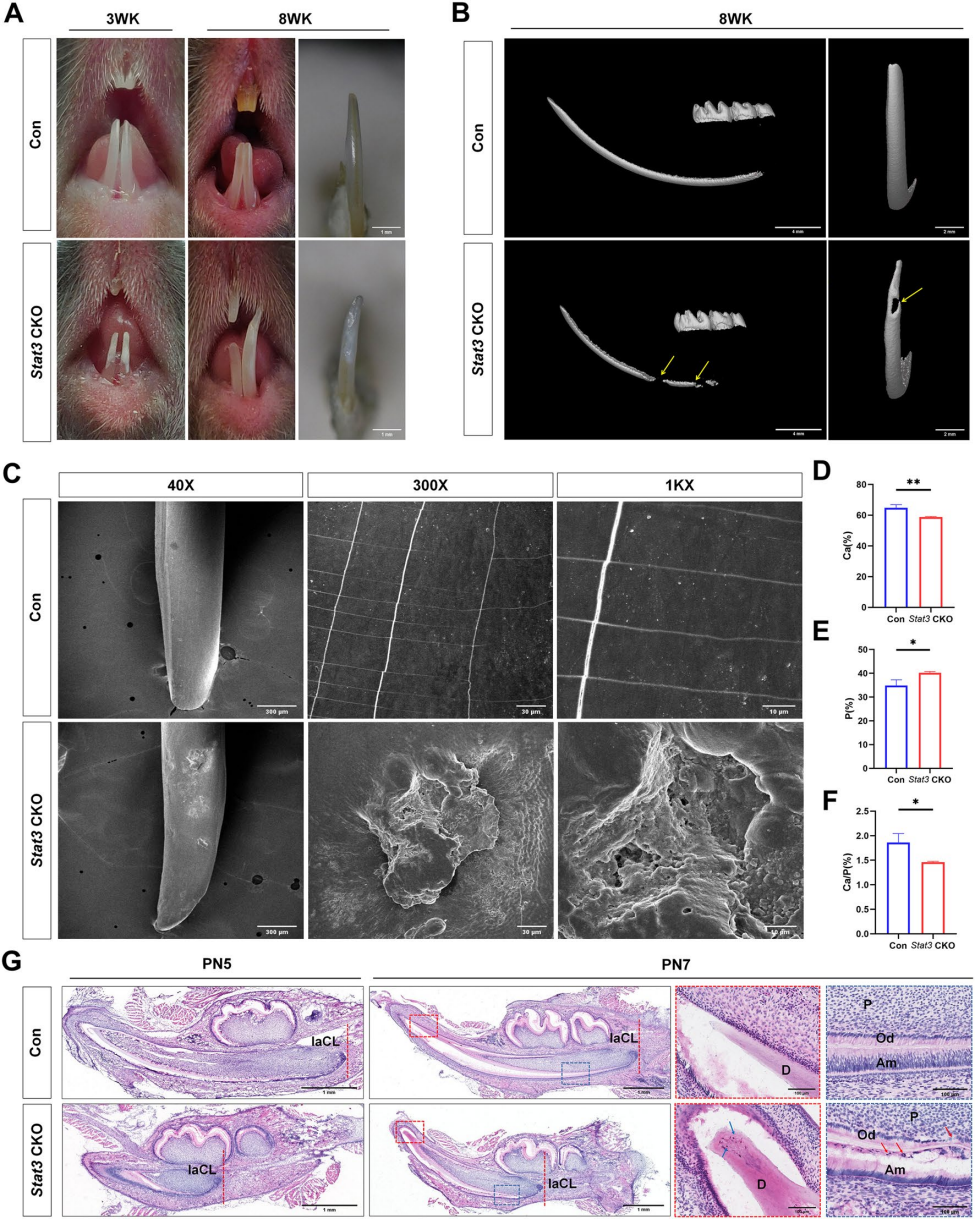
Mineralization defects in incisor enamel of *Stat3* CKO mice. **A** Gross morphology of incisor at 3-weeks-old and 8-weeks-old. In *Stat3* CKO mice, incisors exhibited rough and chalky enamel. **B** µCT reconstruction of *Stat3* CKO mice at 8-weeks-old showed enamel mineralization defects (yellow arrow). **C** SEM and EDS analysis of incisors from 8-week-old mice. Compared with control mice, the enamel surface was irregular with defects in *Stat3* CKO mice, and honeycomb-like micropores could be seen under high magnification. **D**–**F** Quantification of Ca, P, and Ca/P ratios on the labial surfaces of mandibular incisors using energy dispersive spectroscopy. **G** Histological features of developing labia loops of incisor at PN5 and PN7. Developing labial loops of incisor delayed compared with control mice. The red dashed line indicated where laCL is located. Red frames and blue frames indicate the cusp areas and are close to laCL of the mandibular incisors, respectively. At higher magnification, ectopic cells appeared between ameloblasts and dentin in the incisors of *Stat3* CKO mice (red arrow). Osteodentin was observed at the coronal part in *Stat3* CKO mice (blue arrow). *laCL* labial cervical loops, *Am* ameloblast, *Od* odontoblast, *P* pulp, *D* dentin. n = 3, *P < 0.05, **P < 0.01

*Osx*-Cre was expressed only in dental mesenchymal cells but not in dental epithelial cells (Fig. 1). Interestingly, at 3 weeks-old (3WK) and 8 weeks-old (8WK), the incisive enamel of control mice was smooth and yellowish by gross observation. In contrast, *Stat3* CKO mice exhibited rough and chalky enamel (Fig. 2A). Significant enamel defects in incisors of 8WK *Stat3* CKO mice were found by µCT assessment (Fig. 2B, indicated by yellow arrows). However, similar enamel defects were not observed on molar.

To determine enamel mineralization defects caused by loss of Stat3, SEM and EDS analysis were carried out on mandibular incisors. In the control mice, the enamel surface was flat and smooth without enamel defects, or only some tiny cracks and particle deposition were found under high magnification (Fig. 2C). In contrast, the enamel surface of *Stat3* CKO mice were irregular with defects, showing honeycomb-like micropores at high magnification (Fig. 2C). EDS results showed that the Ca content and Ca/P ratio decreased in *Stat3* CKO samples (Fig. 2D–F), indicating that these micropores may be an exchange channels between internal and external substances, which makes it more susceptible to demineralization.

Histological analysis showed that the incisor labial cervical loops (laCL) of *Stat3* CKO mice were developed abnormally and significantly shorter. At PN5, laCL of *Stat3* CKO mice was located proximally to the first molar, whereas laCL of the control mice was located distally to the first molar (Fig. 2G, the position of laCL was indicated by the red dashed line). The same observations were found in PN7 mice. The posterior growth of laCL in *Stat3* CKO mice was always lagging behind the control (Fig. 2G). At higher magnification, ectopic cells appeared between ameloblasts and dentin in the incisors of *Stat3* CKO mice (Fig. 2G, indicated by red arrow).

### Thinned dentin, widened pulp chamber, and shortened roots were found on *Stat3* CKO mice

**Fig. 3:**
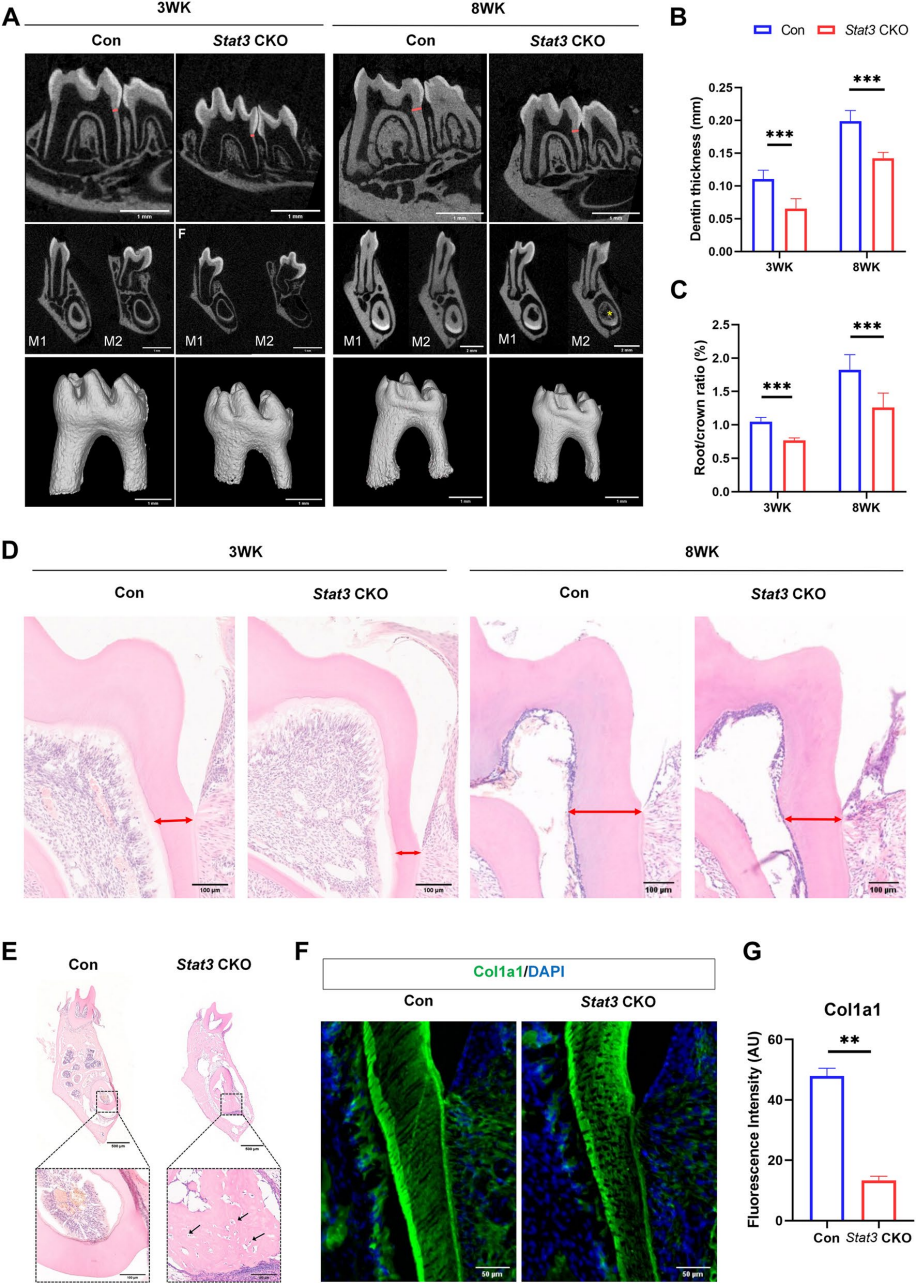
Defects in dentin formation and shortened roots in *Stat3* CKO mice. **A** µCT scans from the mandibular molars of *Stat3* CKO and control mice at 3 and 8WK. *Stat3* CKO mice had thinner dentin thickness in root, larger pulp cavity and shorter root length. Dentin marked in red was used for dentin thickness analysis. A high density shadow was seen in the incisors pulp cavity in the coronal section of mandibular second molars from *Stat3* CKO mice at 8WK. * indicates high-density shadow in the incisor pulp cavity. **B** Quantifications of root dentin thickness of the first molar at 3 and 8WK. **C** Quantifications of root to crown ratio of the distal root of the first molar at 3WK and 8WK. **D** Histological comparison to molar dentin thickness at 3WK and 8WK old. *Stat3* CKO mice showed less thickness in the root dentin than control mice. **E** Osteodentin formation in the mandibular incisors of the *Stat3* CKO mice at 8WK. Black arrows indicated osteodentin. **F** Representative images of Col1a1 immunofluorescent staining in the first mandibular molar from 3WK. **G** The protein expression levels of Col1a1 in odontoblasts of control and *Stat3* CKO mice. n = 3, **P < 0.01, ***P < 0.001

To study the effect of Stat3 on dentin, µCT scans of the mandibles of 3WK and 8WK mice were performed to observe the dentin of molars and incisors. The results showed that dentin thickness decreased, and the pulp chamber enlarged significantly in *Stat3* CKO mice, compared with the control (Fig. 3A, B). Root length of CKO mice was shorter than that of control mice on both 3WK and 8WK animals (Fig. 3A, C). In addition, for *Stat3* CKO mice at 8WK, a high density shadow was observed in the incisors pulp cavity at the coronal section of mandibular second molars (Fig. 3A, indicated by *).

H&E staining also revealed a decreased dentin thickness in *Stat3* CKO mice (Fig. 3D). For the mandibular incisors at PN5 and 8WK, the osteodentin was observed at the coronal dentin and root dentin in *Stat3* CKO mice (Fig. 2G, blue arrows; Fig. 3E, black arrow). Type I collagen (Col1a1) is a mineralized tissue matrix protein secreted by odontoblasts. The expression level of Col1a1 in 3WK mouse odontoblast cells was significantly lower in *Stat3* CKO mice than that in control mice (Fig. 3F, G). In addition, differences in dentin tubules were observed between the two mouse lines. In control mice, dentin tubules were radially arranged from the dentin cells to the enamel, whereas the dentin tubules in *Stat3* CKO mice were not radially arranged and were porous in structure (Fig. 3F).

### The number of proliferating cells in the root mesenchymal region decreased in *Stat3* CKO mice

**Fig. 4:**
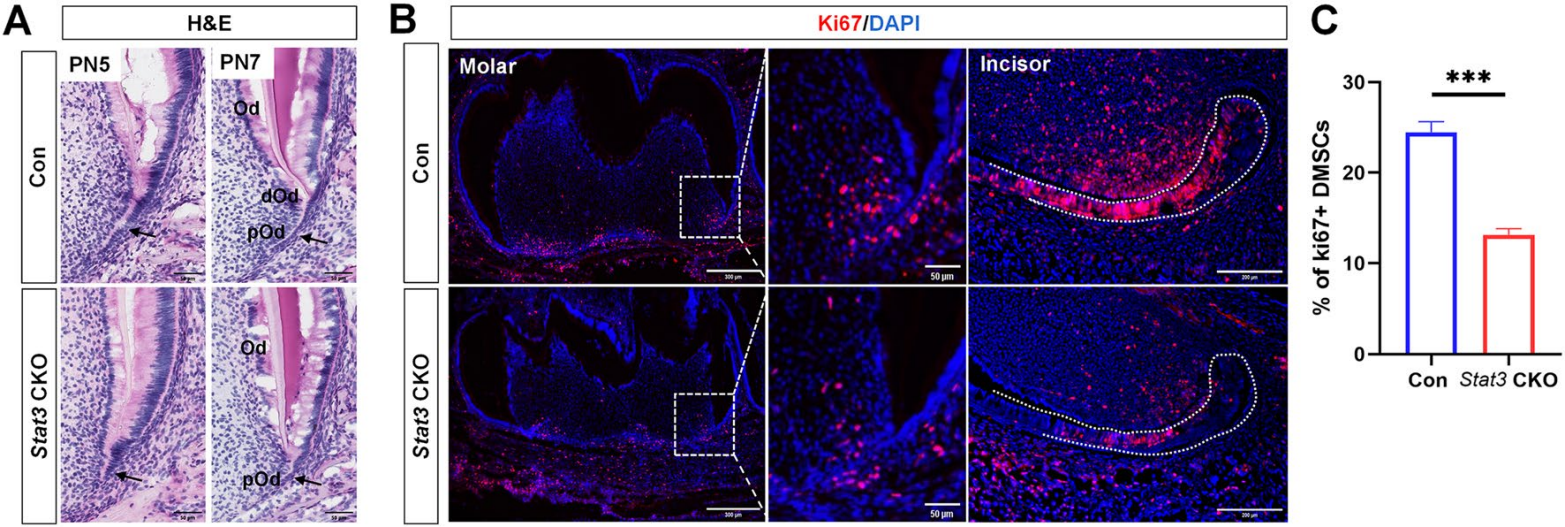
The development of HERS was disrupted in *Stat3* CKO mice. **A** Histological features of developing roots of mandibular first molars at PN5 and PN7. The black arrow indicates HERS. **B** Representative images of Ki67 immunofluorescent staining in the first mandibular molar and incisor from PN7. **C** Quantification of Ki67-positive cells in the mesenchyme of first molars. *Od* odontoblasts, *dOd* differentiating odontoblasts, *pOd* pre-odontoblasts. n = 3, ***P < 0.001

To explore the effects Stat3 on tooth roots, the histological differences in the early development of first molar roots were assessed. At PN5, HERS in control mice extended downward to initiate root formation. In contrast, the extension of HERS was not detected in *Stat3* CKO mice (Fig. 4A, HERS was indicated by black arrows). The difference between control and *Stat3* CKO mice was more pronounced at PN7, where the HRES of control mice extended into the mesenchyme and presented as an elongated epithelial structure (Fig. 4A, HERS is indicated by black arrows). Neatly arranged odontoblasts and adjacent pre-odontoblasts were observed medial to the HERS. In contrast, the HERS of *Stat3* CKO mice had a short and thick structure, lacking, differentiating odontoblasts on the inner side of the HERS.

Since histological differences in HRES between control and *Stat3* CKO mice were evident at PN7. We performed Ki67 staining to analyze cell proliferation in apical region of molar and LaCL of incisor. The results showed that Ki67-positive cells significantly decreased in dental mesenchymal cells of *Stat3* CKO mice (Fig. 4B, C).

### Stat3 may affect dentin formation and root development through β-catenin

**Fig. 5:**
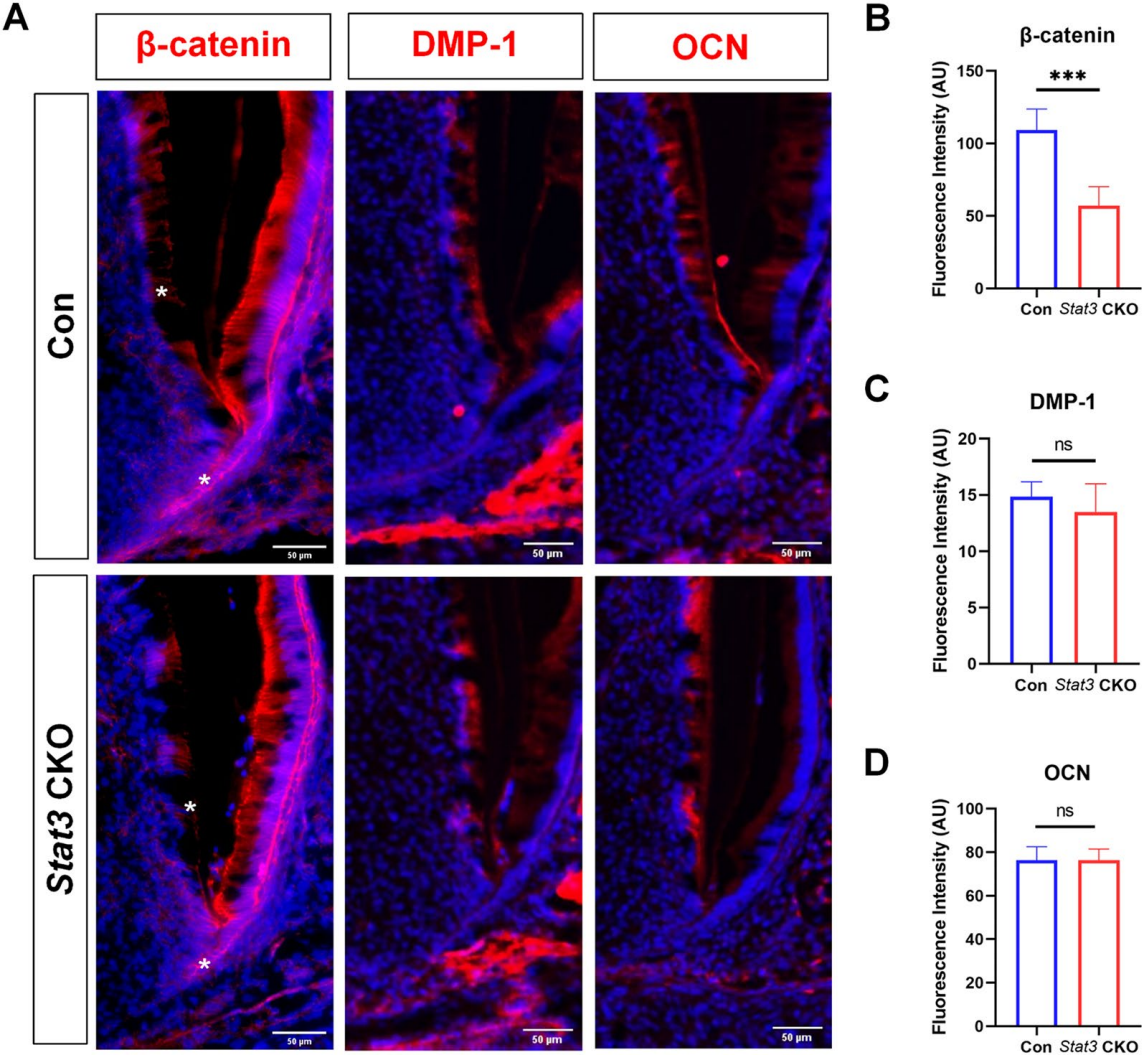
**β**-catenin, DMP-1 and OCN expression in *Stat3* CKO mice.** A** Representative images of β-catenin, DMP-1 and OCN immunofluorescent staining in the first mandibular molar from PN7. *Indicates regions of difference. **B**–**D** The protein expression levels of β-catenin, DMP-1 and OCN in developmental teeth of control and *Stat3* CKO mice. n = 3, ***P < 0.001

Wnt/β-catenin signaling plays multiple roles in various stages of tooth morphogenesis [21]. To explore the possible mechanism of Stat3 on dentin formation, the expressions of β-catenin and odontoblasts related proteins DMP-1 and OCN were analyzed (Fig. 5A). The results showed that DMP-1 and OCN expressions were not significantly different between control and *Stat3* CKO mice (Fig. 5C, D). In *Stat3* CKO mice, β-catenin was expressed in odontoblasts and HERS, whereas the expression was lower than that of control mice (Fig. 5B).

## Discussion

In this study, the effect of Stat3 on the development of dental hard tissues was investigated. *Stat3* CKO mice exhibited remarkable tooth phenotypes characterized by shortened root and thinned dentin in molars, and osteodentin formation in incisor. The abnormalities revealed that Stat3 played an important role in dentin formation. In addition, abnormal enamel mineralization was found in *Stat3* CKO mice, which suggested that Stat3 may affect enamel development through epithelial-mesenchymal interaction.

In this study, *Osx*-*Cre* was specifically expressed in dental mesenchymal cells, but not in ameloblasts and HERS (Fig. 1). Meanwhile, HERS, which was originated from epithelial cells, extended belatedly in *Stat3* CKO mice (Fig. 5A). It’s known that tooth root morphogenesis depends on epithelial-mesenchymal interactions [22]. HERS extends into mesenchyme to guide root formation, and simultaneously induces the differentiation of odontoblasts inside the HERS to form root dentin [23]. Since *Osx-Cre* didn’t target epithelial cells, the delayed root formation in Stat3 CKO mice of the current study may be the result of mesenchyme-orientated Stat3. In addition, the enamel defects in the mandibular incisors of *Stat3* CKO mice were well observed through µCT scanning, which showed rough and chalky enamel. This implied that the enamel formation from endothelial cells was also influenced by Stat3-deficient dental mesenchyme. Similarly, Bin Zhang et al. reported that the loss of Stat3 in dental endothelial cells (*Krt14*-*Cre*; *Stat3*^*fl/fl*^) caused delayed incisor amelogenesis and dentin abnormalities [16]. Signals from the dental mesenchyme have been shown to affect HRES in several mouse knockout studies, further supporting our hypothesis [24–26]. The above data provided convincing evidence to support the importance of the interaction between epithelium and mesenchyme during forming enamel and dentin. However, it is unclear whether abnormalities occur on molar enamel of *Stat3* CKO mice or not. Further studies are required to determine how the loss of Stat3 in dental mesenchymal cells affect the development of these epithelial tissues.

It is important to note that enamel defects were present only in incisors in our results. Also, the incisors have another special phenotype, which is the formation of osteodentin in the incisors. Osteodentin is formed by different stimulus such as mechanical stimulation, infection, and genetic defects [27–29]. In the present study, osteodentin was observed in *Stat3* CKO mice incisor at PN7 (Fig. 2G, blue arrows), a stage before incisor eruption, so that mechanical stimulation can be temporarily excluded as a cause. According to the report, osteodentin was formed in the molar pulps after deleted *Bmp2* using *Osx*-*Cre* [17]. Knockdown *β-catenin* using *Col1*-*Cre*^*ERT2*^ resulted in aberrant pulp calcification in mouse incisors [30]. The above findings suggested that osteodentin in the incisors of *Stat3* CKO mice was most likely to be caused by *Stat3*-deficiency.

In the current study, the incisors of *Stat3* CKO mice had a specific phenotype that differs from molar teeth, which may be related to the specificity of the model. Rodent incisors are special teeth that can grow continuously throughout life and are often used as an ideal model for stem cell research [31, 32]. Molars have an obvious crown and root axis, whereas incisors have no conventional crown or root, enamel was deposited only on the labial side of the incisor. The labial side containing enamel is referred to as the crown analogue, while the remaining lingual side is referred to as the root analogue[33]. Previous studies have reported that some of the signaling networks that regulating tooth organogenesis and regeneration are different in molars and incisors [34]. For example, the incisor mesenchyme was more susceptible to Wnt/β-catenin signaling. Depletion of *Ctnnb1* using *Osr2-Cre *was found the differential responses to Wnt/β-catenin signaling between the incisor and molar germs. This phenomenon might result from the differential expression of Syndecan-1 between the incisor and molar mesenchyme [35]. Although incisor and molar in mice are not exactly the same on development and disease, we found in our study that both incisor root analogue region and molar root showed a decrease in dentin thickness. This may be related to the developmental model of the lingual side of mouse incisor is similar to that of the molar root[36].

In this study, *Stat3* CKO mice showed reduced proliferation of mesenchymal cells at the molar apical region and incisor laCL region (Fig. 4B). A previous study has shown that dental mesenchymal proliferation played an important role in tooth root morphogenesis [37]. Continuous growth of mouse incisor teeth is dependent on populations of stem cells located at the most proximal end of the incisor (often referred as the cervical end) [38, 39]. Stat3 is considered essential in embryonic stem cells and cancer stem cells [40]. Sarper SE et al. suggested that Stat3 phosphorylation regulates epithelial stem cells, which mediate the Runx1-Lgr5 axis to maintain the homeostasis of continued incisor growth [41]. Therefore, we suggest that the shortened root and thinned dentin seen in *Stat3* CKO mice may be related to cell proliferation alterations. It’s tempting to speculate that Stat3 was involved in the regulation of dental stem cells and played an important role in maintaining the continuous growth of incisor.

It is reported recently that bone defects in the Job Syndrome are likely caused by Wnt/β-catenin signaling reduction due to reduced STAT3 activities in bone development, and enhancing Wnt/β-catenin signaling can rescue the bone defects of the *Stat3* mutants [42]. According to the literature, the Wnt/β-catenin signaling pathway participates in all stages of tooth development [21]. Depleted of *β-catenin* in odontoblasts using *OC*-*Cre* resulted in completely disrupted root formation, which is manifested as no dentin formation due to failure of odontoblast differentiation in the root region [24, 26]. The interaction between Stat3 and β-catenin has been widely reported [43–45]. In our study, we found that loss of Stat3 resulted in lower β-catenin expression in HERS and odontoblasts (Fig. 5A, B). It suggested that Stat3 may affect the development of tooth roots and dentin by regulating cell proliferation and differentiation through the β-catenin signaling pathway. Meanwhile, detailed mechanism of Stat3 and β-catenin signaling in root development is a valuable research question to explore in further studies.

In conclusion, our study suggested that Stat3 in Osterix^+^ cells had an important role in dental epithelial and dental mesenchymal interactions during root development. In addition, Stat3 may regulate tooth development through β-catenin signaling. However, these mechanisms require further investigation.

## Data Availability

All datasets used and/or analyzed during the current study are available from the corresponding author on reasonable request.

## Abbreviations

Stat3: Signal transducers and activators of transcription 3
CKO: Conditional knockout
HERS: Hertwig's epithelial root sheath
laCL: Labial cervical loops
OSX: Osterix
DMP-1: Dentine matrix protein 1
Col1a1: Collagen I
OCN: Osteocalcin
